# Molecular Basis Underlying Hepatobiliary and Renal Excretion of Phenolic Acids of *Salvia miltiorrhiza* Roots (Danshen)

**DOI:** 10.3389/fphar.2022.911982

**Published:** 2022-05-10

**Authors:** Jun-Lan Lu, Xue-Shan Zeng, Xin Zhou, Jun-Ling Yang, Ling-Ling Ren, Xin-Yu Long, Feng-Qing Wang, Olajide E. Olaleye, Nan-Nan Tian, Ya-Xuan Zhu, Jia-Jia Dong, Wei-Wei Jia, Chuan Li

**Affiliations:** ^1^ Graduate School, Tianjin University of Traditional Chinese Medicine, Tianjin, China; ^2^ State Key Laboratory of Drug Research, Shanghai Institute of Materia Medica, Chinese Academy of Sciences, Shanghai, China; ^3^ School of Pharmacy, University of Chinese Academy of Sciences, Beijing, China; ^4^ College of Pharmacy, Nanjing University of Chinese Medicine, Nanjing, China

**Keywords:** Danshen, Salvia miltiorrhiza, phenolic acid, hepatic transporter, renal transporter

## Abstract

Phenolic acids are cardiovascular constituents (originating from the Chinese medicinal herb *Salvia miltiorrhiza* root/Danshen) of DanHong and many other Danshen-containing injections. Our earlier pharmacokinetic investigation of DanHong suggested that hepatic and/or renal uptake of the Danshen compounds was the crucial steps in their systemic elimination. This investigation was designed to survey the molecular basis underlying hepatobiliary and renal excretion of the Danshen compounds, i.e., protocatechuic acid, tanshinol, rosmarinic acid, salvianolic acid D, salvianolic acid A, lithospermic acid, and salvianolic acid B. A large battery of human hepatic and renal transporters were screened for transporting the Danshen compounds and then characterized for the uptake kinetics and also compared with associated rat transporters. The samples were analyzed by liquid chromatography/mass spectrometry. Because the Danshen phenolic acids are of poor or fairly good membrane permeability, their elimination *via* the liver or kidneys necessitates transporter-mediated hepatic or renal uptake from blood. Several human transporters were found to mediate hepatic and/or renal uptake of the Danshen compounds in a compound-molecular-mass-related manner. Lithospermic acid and salvianolic acid B (both >500 Da) underwent systemic elimination, initiated by organic anion-transporting polypeptide (OATP)1B1/OATP1B3-mediated hepatic uptake. Rosmarinic acid and salvianolic acids D (350–450 Da) underwent systemic elimination, initiated by OATP1B1/OATP1B3/organic anion transporter (OAT)2-mediated hepatic uptake and by OAT1/OAT2-mediated renal uptake. Protocatechuic acid and tanshinol (both <200 Da) underwent systemic elimination, initiated by OAT1/OAT2-mediated renal uptake and OAT2-mediated hepatic uptake. A similar scenario was observed with the rat orthologs. The investigation findings advance our understanding of the disposition of the Danshen phenolic acids and could facilitate pharmacokinetic research on other Danshen-containing injections.

## Introduction

*Salvia miltiorrhiza* roots (Danshen in Chinese) are a commonly used Chinese cardiovascular herb which, alone or in combination with other medicinal herbs, is formulated for oral or intravenous administration. Hydrophilic phenolic acids and lipophilic diterpene quinones are believed to be responsible for most of the cardiovascular effects of Danshen (Li et al., 2018; Ren et al., 2019). DanHong injection is prepared from a 3:1 mixture of Danshen and *Carthamus tinctorius* flowers (Honghua) (Feng et al., 2019). It is an injectable solution available as a sterile, nonpyrogenic parenteral dosage form for intravenous administration. DanHong is approved by the Chinese National Medical Products Administration (NMPA) for the treatment of atherosclerotic coronary artery disease and acute ischemic stroke. In a recent adaptive, 31-center, double-blind, randomized controlled trial in 920 patients with stable angina, treatment with 14-days DanHong significantly reduced the number of angina episodes and improved angina-specific health status for at least 90 days, particularly for patients with severe angina, faster heart rate, or diabetes (Liu et al., 2021). It is reassuring that no substantial harm was observed in the clinical trial.

**FIGURE 1:**
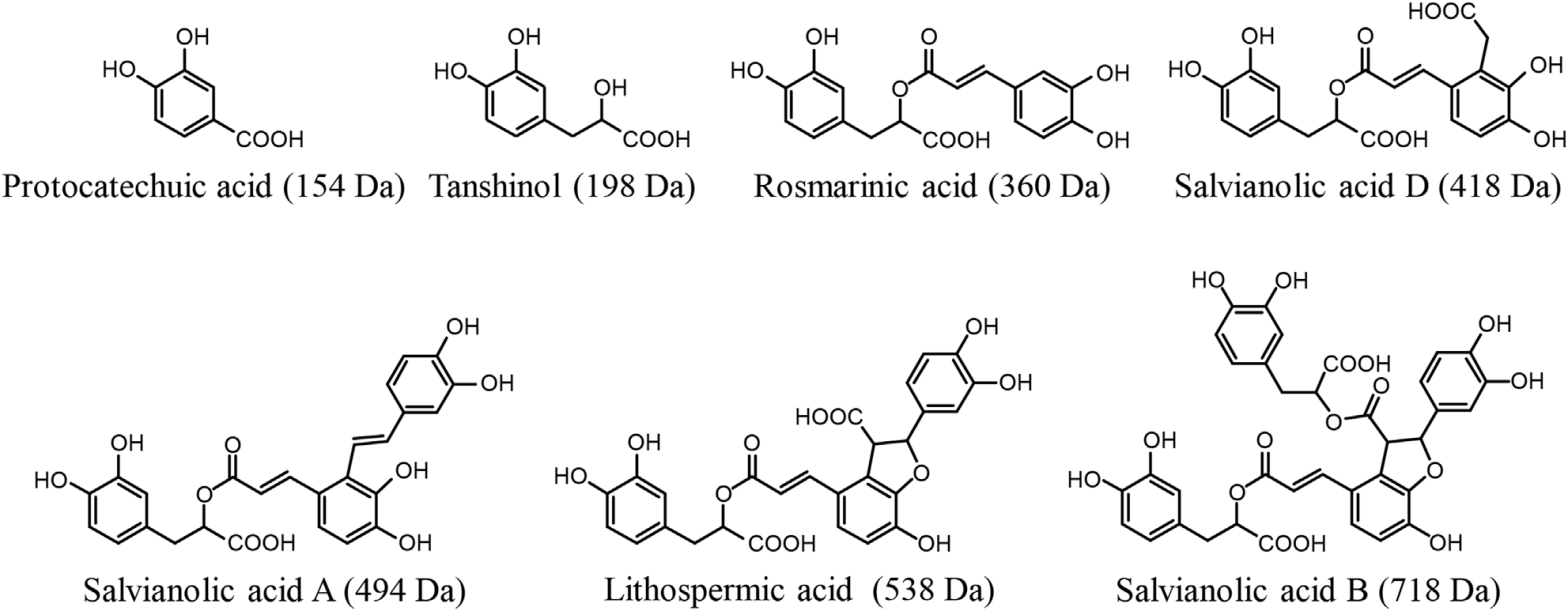
Chemical structures of Danshen phenolic acids.

Due to the aqueous extraction for preparing DanHong, the constituents originating from Danshen were the hydrophilic acids, rather than the lipophilic diterpene quinones. The major Danshen hydrophilic phenolic acids present in DanHong are caffeic acid derivatives, occurring as monomers (tanshinol), dimers (rosmarinic acid and salvianolic acid D), trimers (salvianolic acid A and lithospermic acid), and tetramers (salvianolic acid B). In addition, these compounds contain one or more catechol moieties and have one or more carboxylic acid groups (Jiang et al., 2005). Protocatechuic aldehyde is also a major Danshen hydrophilic compound in DanHong; it is a catechol compound but has no carboxy group. In our earlier multi-compound pharmacokinetic investigation of DanHong, these Danshen compounds were evaluated for systemic exposure in humans (who intravenously received the herbal injection) and for elimination from the systemic circulation (Li et al., 2015). After dosing DanHong, metabolism of the Danshen phenolic acids occurs mainly in the liver and comprises methylation, glucuronidation, and sulfation. Given that all these phenolic acids have poor or fairly good membrane permeability, hepatic uptake, which is the prerequisite for their metabolism, needs to be mediated by some transporters. Meanwhile, the kidneys can also compete with the liver for elimination of some Danshen phenolic acids. Renal excretion of these Danshen compounds appears to involve tubular secretion, which needs transporter(s) to mediate the renal uptake. After dosing DanHong, protocatechuic aldehyde is extensively transformed into protocatechuic acid by hepatic aldehyde dehydrogenase, and this metabolite is a phenolic acid, which is eliminated as unchanged and metabolized forms by renal excretion. This investigation was designed to survey the molecular basis of the preceding hepatobiliary and renal excretion of the compounds (Figure 1). The investigation findings advance our understanding of disposition of Danshen phenolic acids. Given that these Danshen phenolic acids are also major active compounds of many other Danshen-containing herbal injections (Supplementary Table S1), the results of the current investigation could facilitate pharmacokinetic research on these herbal injections.

## Materials and Methods

### Chemicals and Reagents

The test Danshen compounds protocatechuic acid, tanshinol, rosmarinic acid, salvianolic acid D, salvianolic acid A, lithospermic acid, and salvianolic acid B were obtained from the Tongtian Biotechnology (Shanghai, China) and their purity exceeded 98%. The positive substrates for testing validity of transporter systems, i.e., estradiol-17β-D-glucuronide (E_2_17βG), estrone-3-sulfate (E_1_S), prostaglandin F_2α_ (PGF_2α_), tetraethylammonium (TEA), taurocholic acid (TCA), para-aminohippuric acid (PAH), glycylsarcosine (GS), methotrexate (MTX), and glycyrrhizin (GL) were obtained from Sigma-Aldrich (St. Louis, MO, United States). Adenosine 5′-triphosphate (ATP; disodium salt hydrate) and other reagents were obtained from Sinopharm Chemical Reagent Co., Ltd. (Shanghai, China).

Human embryonic kidney 293 (HEK-293) cells were obtained from the American Type Culture Collection (Manassas, VA, United States). Full open reading frames of cDNA for human organic anion-transporting polypeptide (OATP) 1B1, OATP1B3, OATP2B1, organic anion transporter (OAT) 1, OAT2, OAT3, OAT4, organic cation transporter (OCT) 1, OCT2, OCT3, Na^+^/taurocholate cotransporting polypeptide (NTCP), peptide transporter (PEPT) 1, and PEPT2 and rat Oatp1a1, Oatp1b2, Oat1, Oat2, Oat3, Oct1, Oct2, Oct3, carnitine/organic cation transporter (Octn) 2, Ntcp, Pept1, and Pept2 were synthesized and subcloned into pcDNA 3.1 (+) expression vectors by Invitrogen Life Technologies (Shanghai, China). Prior to the study, all expression plasmids were sequence-verified according to their GeneBank accession numbers, i.e., NM_006446 (human OATP1B1), NM_019844 (human OATP1B3), NM_007256 (OATP2B1), NM_004790 (OAT1), NM_006672 (OAT2), NM_004254 (OAT3), NM_018484 (OAT4), NM_003057 (OCT1), NM_003058 (OCT2), NM_021977 (OCT3), and NM_003049 (NTCP) and NM_017111 (Oatp1a1), NM_031650 (Oatp1b2), NM_017224 (Oat1), NM_053537 (Oat2), NM_031332 (Oat3), NM_012697 (Oct1), NM_031584 (Oct2), NM_019230 (Oct3), NM_019269 (Octn2), and NM_017047 (Ntcp).

Inside-out membrane vesicles (5 mg protein/mL), prepared from insect cells expressing human multidrug resistance-associated protein (MRP) 2, MRP3, MRP4, breast cancer resistance protein (BCRP), bile salt export pump (BSEP), and multidrug resistance protein (MDR) one and rat Mrp2, Mrp4, Bcrp, and Bsep, and vesicles (5 mg protein/mL; prepared from the insect cells expressing no ABC transporter; serving as negative control) were obtained from Genomembrane (Kanazawa, Japan).

### Cell Culture and Transfection

Cell cultures and transfection were performed as described previously (Jia et al., 2015; Dong et al., 2018). Briefly, HEK-293 cells were grown, at 37°C and 5% CO_2_, in Dulbecco’s modified Eagle’s medium, which was fortified with 10% fetal bovine serum, 1% minimal essential medium nonessential amino acids, and 1% antibiotic-antimycotic solution. A day before transfection, cells were seeded onto 24-well poly-d-lysine-coated plates at a density of 2 × 10^5^ cells per well. After incubation at 37°C and 5% CO_2_ for 24 h, the expression plasmid and the empty vector were introduced separately into the HEK293 cells with Lipofectamine 2000 transfection reagent (Invitrogen, Carlsbad, CA, United States), according to the manufacturer’s protocol to yield transporter-expressing cells and mock cells, respectively. Before use, the transfected cells were validated functionally using the positive substrate GL (OATP1B1, OATP1B3, and Oatp1b2), E_2_17βG (for Oatp1a1), PAH (OAT1 and Oat1), PGF_2α_ (OAT2 and Oat2), E_1_S (OATP2B1, OAT3, OAT4, and Oat3), TEA (OCT1, OCT2, OCT3, Oct1, Oct2, Oct3, and Octn2), TCA (NTCP and Ntcp), and GS (PEPT1, PEPT2, Pept1, and Pept2) (Jia et al., 2015; Jiang et al., 2015; Dong et al., 2018).

### Cellular Transport Assays

Transport studies were performed 48 h after the transfection. The test Danshen compounds, i.e., protocatechuic acid, tanshinol, rosmarinic acid, salvianolic acid D, salvianolic acid A, lithospermic acid, and salvianolic acid B at 100 µM final concentration each, were separately incubated with the transfected cells for 10 min. Krebs-Henseleit buffer was used for preincubation, incubation, and cell washing, except for the transporters PEPT1, PEPT2, Pept1, and Pept2 using buffer A (containing 145 mM NaCl, 3 mM KCl, 1 mM CaCl_2_, 0.5 mM MgCl_2_, 5 mM glucose, and 5 mM HEPES, pH 7.4) for preincubation and cell washing and buffer B (containing 145 mM NaCl, 3 mM KCl, 1 mM CaCl_2_, 0.5 mM MgCl_2_, 5 mM glucose, and 5 mM MES, pH 6.0) for incubation. The transport rate (pmol/mg protein/min) of the test Danshen phenolic acids was calculated using Equation (1):

$$\text{Transport}=\left(C_{\text{L}}\text{×}V_{\text{L}}\right)/T/W_{\text{L}}$$ (1)

where *C*_L_, *V*_L_, *T*, and *W*_L_ are the concentration of the test Danshen compound in the cellular lysate (μM), the volume of the lysate (μL), the incubation time (10 min), and the protein amounts measured in the lysate (mg), respectively. Differential uptake between the transfected cells and mock cells was defined as net transport ratio (Transport_TC_/Transport_MC_ ratio), where Transport_TC_ and Transport_MC_ are the transport rates of a test Danshen compound into transfected and mock cells, respectively. A net transport ratio >3, with a statistically significant difference between Transport_TC_ and Transport_MC_, demonstrated enough substrate activity in the screen to warrant determination of the compound for the Michaelis constant (*K*_m_), maximum velocity (*V*_max_), and intrinsic clearance (CL_int_). The determination was performed under linear uptake conditions by incubation for 5 min. Table 1 summarizes the final concentrations of the test Danshen compounds for different transporters. All the incubation samples were analyzed by liquid chromatography/mass spectrometry.

**TABLE 1:** Final concentrations of Danshen phenolic acids used in the cellular uptake kinetic study.

Danshen phenolic compound	SLC transporter	Final concentration (µM)
Protocatechuic acid	Human OAT1	7.81–500
	Human OAT2	15.6–1,000
	Rat Oat1	25.0–600
	Rat Oat2	15.6–1,000
	Rat Oat3	25.0–600
Tanshinol	Human OAT1	15.6–1,000
	Human OAT2	312–10,000
	Human OAT3	156–5,000
	Human OAT4	312–10,000
	Rat Oat1	25.0–600
	Rat Oat2	100–3,200
	Rat Oat3	312–10,000
Rosmarinic acid	Human OATP1B1	62.5–4,000
	Human OATP1B3	62.5–4,000
	Human OAT1	6.25–400
	Human OAT2	12.5–600
	Human OAT4	31.2–2,000
	Rat Oatp1b2	100–6,400
	Rat Oat1	12.5–400
	Rat Oat2	25.0–600
	Rat Oat3	12.5–400
Salvianolic acid D	Human OATP1B1	50.0–1,600
	Human OATP1B3	50.0–3,200
	Human OAT1	6.25–400
	Human OAT2	25.0–600
	Rat Oatp1b2	50.0–3,200
	Rat Oat1	6.25–400
	Rat Oat2	25.0–600
Salvianolic acid A	Rat Oatp1b2	12.5–400
Lithospermic acid	Human OATP1B1	12.5–800
	Human OATP1B3	12.5–800
	Rat Oatp1b2	6.25–600
Salvianolic acid B	Human OATP1B1	3.13–200
	Human OATP1B3	3.13–200
	Rat Oatp1b2	3.13–200

### Vesicular Transport Assays

Inside-out membrane vesicles expressing one of the ABC transporters MRP2, MRP3, MRP4, BCRP, BSEP, MDR1, Mrp2, Mrp4, Bcrp, and Bsep were used to assess the transport of the test Danshen compound, i.e., protocatechuic acid, tanshinol, rosmarinic acid, salvianolic acid D, salvianolic acid A, lithospermic acid, and salvianolic acid B, using a rapid filtration method (Jiang et al., 2015; Dong et al., 2018). Before use, membrane vesicles expressing MRP2, MRP3, MRP4, Mrp2, or Mrp4 were functionally validated using E_2_17βG, those expressing BCRP or Bcrp using MTX, those expressing BSEP or Bsep using TCA, and those expressing MDR1 using GL. The test Danshen compounds at 100 µM final concentration were separately incubated with the membrane vesicles for 10 min. Buffer C (50 mM MOPS-tris, 70 mM KCl, 7.5 mM MgCl_2_, pH 7.0) was used for preincubation and incubation, while buffer D (40 mM MOPS-tris, 70 mM KCl, pH 7.0) was used for vesicle washing. The transport rate (pmol/mg protein/min) of the test Danshen compound was calculated using Equation (2):

$$\text{Transport}=\left(C_{\text{V}}\text{×}V_{\text{V}}\right)/T/W_{\text{V}}$$ (2)

where *C*_V_, *V*_V_, *T*, and *W*_V_ represent concentration of the compound in vesicular lysates supernatant (μM), volume of the lysates (μL), incubation time (10 min), and amount of vesicle protein amount per well (0.05 mg), respectively. Differential transport of the test Danshen compound between ATP-containing vesicles and AMP-containing vesicles was defined as net transport ratio (Transport_ATP_/Transport_AMP_ ratio). Based on the experience in our laboratory, a net transport ratio >3, with a statistically significant difference between Transport_ATP_ and Transport_AMP_, often suggests a positive result. All the incubation samples were analyzed by liquid chromatography/mass spectrometry.

### Liquid Chromatography/Mass Spectrometry-Based Bioanalytical Assays

Validated bioanalytical assays were used to measure the test Danshen compounds in cell- and vesicle-based biomatrices. Analyses were performed on a TSQ Vantage mass spectrometer (Thermo Fisher, San Jose, CA, United States) interfaced *via* a HESI source with an Agilent 1290 infinity LC system (Waldbronn, Germany). The chromatographic separation was achieved on Phenomenex Gemini 5-μm C_18_ column (50 mm × 2.0 mm i.d, Torrance, CA, United States). The mobile phases, which consisted of solvent A (water/methanol, 99:1, v/v, containing 0.15% formic acid) and solvent B (water/methanol, 1:99, v/v, containing 0.15% formic acid), was delivered at 0.3 ml/min. A 6-min gradient elution method was used as follows: 0–3.5 min, from 1% solvent B to 40% solvent B; 3.5–4.5 min, at 98% solvent B; and 4.6–6 min, at 1% solvent B. The mass spectrometry measurement was performed in the negative ion mode with precursor-product ion pairs for selected-reaction-monitoring of protocatechuic acid, tanshinol, rosmarinic acid, salvianolic acid D, salvianolic acid A, lithospermic acid, and salvianolic acid B at *m/z* 153→108, 197→123, 359→133, 417→197, 493→185, 537→185, and 717→321, respectively. Matrix-matched calibration curves for quantification of these test Danshen compounds (4.1, 12.3, 37, 111, 333, and 500 nM) were constructed using weighted (1/*X*) linear regression of the peak area (*Y*) against the corresponding nominal analyte concentration (*X*, nM). The sample preparation was performed using methanol-based protein precipitation and centrifugation; the supernatant was analyzed by liquid chromatography/mass spectrometry. Although no internal standard was used, assay validation, implemented according to the European Medicines Agency Guideline on bioanalytical method validation (2012; www.ema.europa.eu), demonstrated that the developed assays were reliable and reproducible for the intended use. The assays’ lower limit of quantification was 4.1 nM for all the test Danshen compounds. The intra-batch accuracy and precision were within 94.1–105% and 2.41–10.1%, respectively, while associated inter-batch values were within 86.6–104% and 2.91–11.3%, respectively. The coefficients of variation reflecting matrix effects on assays were 2.23–10.7%. The analyte stability under conditions mimicking the analytical process was also evaluated, including storage at 24°C for 3 h, storage at 8°C for 24 h, and three freeze-and-thaw cycles. The analytes were stable under the test conditions, as indicated by the measured mean concentrations fluctuating within 86.9–113% of the nominal concentrations. All the preceding validation results were within the acceptable ranges.

Measurement of E_2_17βG, E_1_S, PGF_2α_, TEA, TCA, PAH, GS, MTX, and GL was achieved using liquid chromatography/mass spectrometry-based methods described previously (Jia et al., 2015; Dong et al., 2018).

### Data Processing

GraFit software (version 5.0; *Erithacus* Software Ltd, Surrey, United Kingdom) was used to determine the *K*_m_ and *V*_max_ values by nonlinear regression analysis of initial transport rates as a function of substrate concentration. *K*_m_ and *V*_max_ values of the test Danshen compound were calculated using the following Equation (3):

$$V=V_{\text{max}}\text{·}S/\left(K_{\text{m}}+S\right)+P_{\text{dif}}\text{·}S$$ (3)

where *V* is the initial transport of the test Danshen compound in transfected cells (pmol/min/mg protein), *V*_max_ is the maximal transport rate (pmol/min/mg protein), *S* is the concentration of the test Danshen compound (μM) and *P*_dif_ is the clearance of the test Danshen compound *via* passive diffusion in mock cells.

All data are expressed as the mean ± standard deviation. Statistical analysis was performed using SPSS Statistics Software (version 19.0; IBM, Chicago, IL, United States). A value of *p* < 0.05 was considered to be the minimum level of statistical significance.

## Results and Discussion

### Transport of Danshen Phenolic Acids by Human and Rat Hepatic Transporters

**FIGURE 2:**
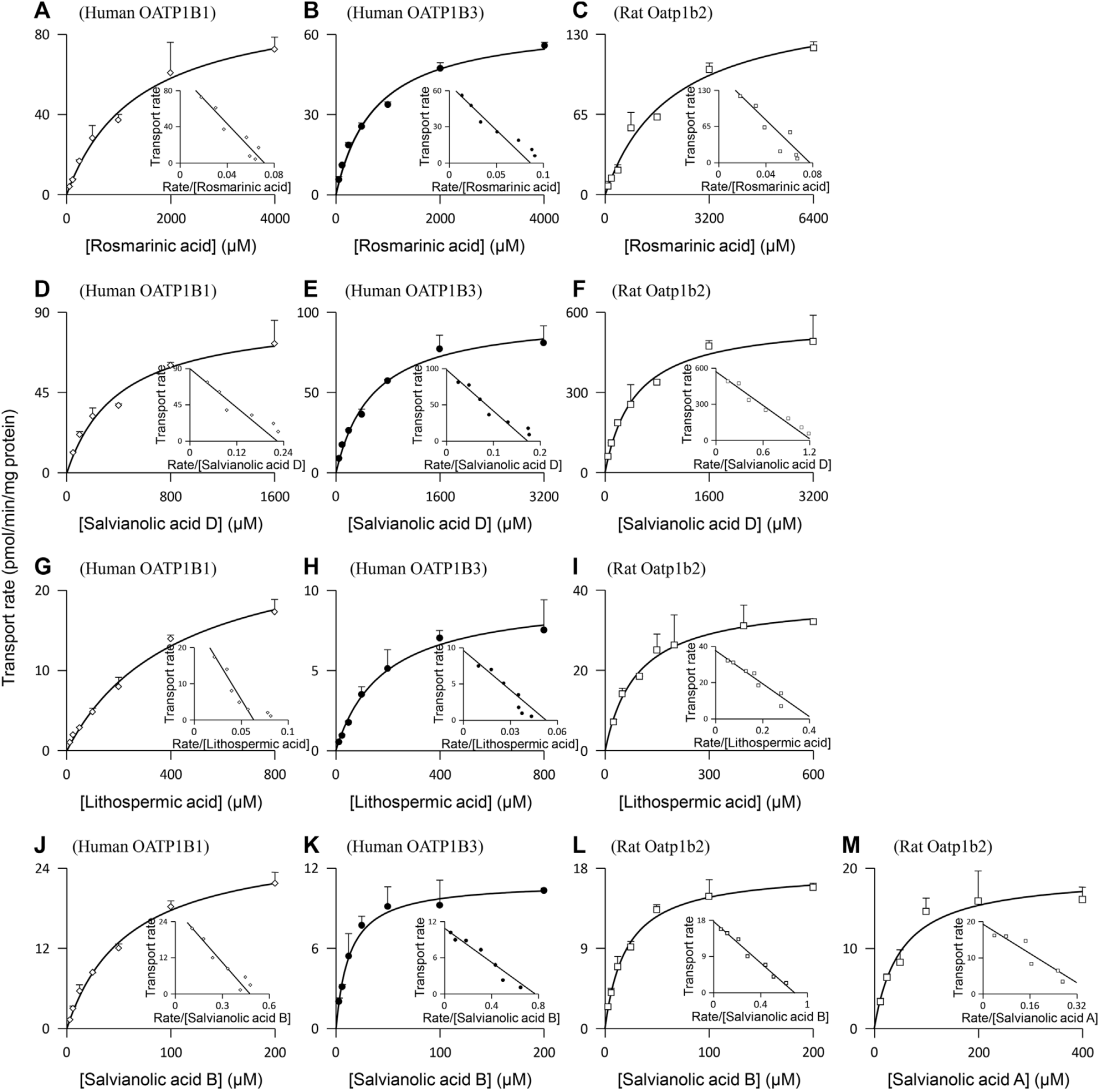
Representative kinetic plots of transport versus substrate concentration for cellular uptake of Danshen phenolic acids [**(A–C)**, rosmarinic acid; **(D–F)**, salvianolic acid D; **(G–I)**, lithospermic acid; **(J–L)**, salvianolic acid B; and **(M)**, salvianolic acid A] mediated by the human and rat hepatic SLC transporters. The *K*_m_, *V*_max_, and CL_int_ values are shown in Table 3, and they represent the means ± standard deviations (*n* = 3). The final concentrations of the test Danshen compounds in the cellular uptake kinetic study are summarized in Table 1.

Rosmarinic acid (molecular mass: 360 Da), salvianolic acid D (418 Da), lithospermic acid (538 Da), and salvianolic acid B (718 Da) were found to be substrates of the human hepatic sinusoidal uptake solute carrier (SLC) transporters OATP1B1 and OATP1B3; their net transport ratios are summarized in Table 2. Figure 2 shows their saturable cellular uptake with *K*_m_, *V*_max_, and CL_int_ values summarized in Table 3. Meanwhile, protocatechuic acid (154 Da), tanshinol (198 Da), rosmarinic acid, and salvianolic acid D were substrates of OAT2 (another human hepatic sinusoidal uptake SLC transporter), with their net transport ratios shown in Table 2 and associated *K*_m_, *V*_max_, and CL_int_ values in Figure 2 and Table 3. The Danshen phenolic acids were not substrates of other human hepatic sinusoidal uptake SLC transporters. Recently, Cao et al. (2019) reported salvianolic acid B as a substrate of human OATP1B1. In the current investigation, we found this Danshen phenolic acid to be also a substrate of human OATP1B3. In addition, salvianolic acid B and lithospermic acid were substrates of human MRP2, a hepatic canalicular efflux ABC transporter, while salvianolic acid D was a substrate of human MRP4, a hepatic sinusoidal efflux ABC transporter. No other human hepatic ABC transporter was found to mediate transport of the Danshen compounds. Despite being closely structure-related to the other Danshen phenolic acids, salvianolic acid A (494 Da) was not a substrate of OATP1B1, OATP1B3, OAT2, or any other test hepatic sinusoidal uptake SLC transporter; it was also not a substrate of hepatic canalicular or sinusoidal efflux ABC transporter. Regarding protocatechuic acid (154 Da) and tanshinol (198 Da), these Danshen phenolic acids had little substrate activity at human hepatic SLC or ABC transporters when screened at 100 μM final concentration (Table 2).

**TABLE 2:** Net transport ratios of Danshen phenolic acids at 100 μM final concentration by human and rat hepatic transporters.

Transporter	Transport_TC_/Transport_MC_ ratio for the SLC transporter or transport_ATP_/Transport_AMP_ ratio for the ABC transporter							
	Positive substrate	Protocatechuic acid	Tanshinol	Rosmarinic acid	Salvianolic acid D	Salvianolic acid A	Lithospermic acid	Salvianolic acid B
MW (Da)		154	198	360	418	494	538	718
Human hepatic sinusoidal uptake SLC transporters								
OATP1B1	79.5 ± 1.3 (GL)	0.83 ± 0.14	1.04 ± 0.13	4.00 ± 0.43*	5.38 ± 0.83*	1.21 ± 0.33	3.99 ± 0.24*	5.16 ± 1.30*
OATP1B3	33.1 ± 0.5 (GL)	0.75 ± 0.09	0.94 ± 0.21	7.28 ± 0.58*	5.83 ± 0.49*	1.02 ± 0.31	8.02 ± 0.52*	16.2 ± 0.6*
OATP2B1	41.1 ± 1.5 (E_1_S)	1.36 ± 0.15	1.45 ± 0.46	1.69 ± 0.71	2.27 ± 1.16	2.45 ± 0.34	1.16 ± 0.27	1.32 ± 0.33
OAT2	70.2 ± 6.6 (PGF_2α_)	417 ± 23*	74.2 ± 13.9*	716 ± 86*	314 ± 44*	2.25 ± 0.47	1.51 ± 0.27	1.04 ± 0.09
OCT1	4.58 ± 0.26 (TEA)	1.09 ± 0.33	0.94 ± 0.11	1.46 ± 0.57	1.04 ± 0.24	1.04 ± 0.32	1.56 ± 0.26	1.03 ± 0.07
OCT3	4.45 ± 0.27 (TEA)	0.94 ± 0.24	0.84 ± 0.32	0.75 ± 0.23	0.94 ± 0.32	1.31 ± 0.09	0.84 ± 0.15	0.75 ± 0.38
NTCP	144 ± 30 (TCA)	1.26 ± 0.15	0.79 ± 0.14	2.76 ± 0.36	2.09 ± 0.36	0.93 ± 0.11	2.44 ± 0.13	2.88 ± 0.21
Human hepatic sinusoidal efflux ABC transporters								
MRP3	6.79 ± 1.49 (E_2_17βG)	1.25 ± 0.08	1.71 ± 0.41	1.38 ± 0.01	1.35 ± 0.30	1.54 ± 0.23	1.72 ± 0.28	1.47 ± 0.33
MRP4	6.17 ± 0.92 (E_2_17βG)	1.25 ± 0.14	1.57 ± 0.06	1.99 ± 0.08	5.27 ± 0.51*	1.56 ± 0.05	1.36 ± 0.14	1.99 ± 0.06
Human hepatic canalicular efflux ABC transporters								
MRP2	7.23 ± 1.04 (E_2_17βG)	0.94 ± 0.31	0.85 ± 0.12	1.13 ± 0.34	1.51 ± 0.73	1.42 ± 0.31	5.02 ± 1.67*	6.21 ± 1.32*
BCRP	4.45 ± 0.67 (MTX)	1.04 ± 0.19	2.55 ± 0.91	1.12 ± 0.55	2.22 ± 0.61	1.12 ± 0.35	1.12 ± 0.31	1.54 ± 0.78
BSEP	4.03 ± 0.89 (TCA)	0.99 ± 0.21	0.93 ± 0.25	1.03 ± 0.09	1.19 ± 0.35	1.32 ± 0.26	0.83 ± 0.20	1.43 ± 0.32
MDR1	5.13 ± 0.87 (GL)	0.93 ± 0.04	1.43 ± 0.27	1.04 ± 0.18	0.92 ± 0.23	1.26 ± 0.05	1.26 ± 0.09	1.66 ± 0.12
Rat hepatic sinusoidal uptake SLC transporters								
Oatp1a1	17.9 ± 2.3 (E_2_17βG)	1.66 ± 0.77	1.67 ± 0.66	1.45 ± 0.57	2.45 ± 1.88	2.24 ± 0.67	1.41 ± 0.67	1.91 ± 0.42
Oatp1b2	121 ± 2 (GL)	1.32 ± 0.45	1.45 ± 0.32	17.0 ± 2.8*	16.0 ± 0.8*	7.52 ± 1.31*	11.6 ± 0.7*	30.0 ± 0.6*
Oat2	24.3 ± 3.3 (PGF_2α_)	163 ± 30*	17.2 ± 2.8*	334 ± 18*	50.5 ± 10.7*	1.41 ± 0.26	1.36 ± 0.33	0.92 ± 0.22
Oct1	8.16 ± 2.40 (TEA)	1.19 ± 0.41	0.89 ± 0.34	1.56 ± 0.13	1.23 ± 0.54	1.13 ± 0.19	1.18 ± 0.06	0.66 ± 0.17
Oct3	3.90 ± 0.88 (TEA)	0.85 ± 0.11	1.01 ± 0.11	0.74 ± 0.24	0.93 ± 0.15	1.19 ± 0.14	0.81 ± 0.13	0.84 ± 0.31
Octn2	9.85 ± 0.74 (TEA)	0.91 ± 0.17	0.82 ± 0.19	1.14 ± 0.04	1.12 ± 0.22	0.95 ± 0.44	0.95 ± 0.11	1.25 ± 0.21
Ntcp	134 ± 15 (TCA)	1.04 ± 0.27	0.95 ± 0.16	1.68 ± 0.25	1.49 ± 0.23	0.84 ± 0.13	1.71 ± 0.15	1.90 ± 0.02
Rat hepatic sinusoidal efflux ABC transporters								
Mrp4	7.37 ± 1.32 (E_2_17βG)	1.17 ± 0.06	1.81 ± 0.15	4.79 ± 0.38*	11.1 ± 0.2*	2.09 ± 0.25	1.46 ± 0.01	2.15 ± 0.32
Rat hepatic ABC canalicular efflux ABC transporters								
Mrp2	3.54 ± 0.32 (E_2_17βG)	0.74 ± 0.13	0.75 ± 0.20	1.24 ± 0.45	2.21 ± 0.89	1.80 ± 0.81	1.83 ± 0.45	4.43 ± 1.12*
Bcrp	20.5 ± 3.2 (MTX)	0.94 ± 0.21	0.83 ± 0.23	1.45 ± 0.32	5.54 ± 1.03*	1.31 ± 0.04	1.27 ± 0.20	1.13 ± 0.14
Bsep	5.14 ± 0.88 (TCA)	1.13 ± 0.17	1.20 ± 0.25	1.39 ± 0.36	1.09 ± 0.23	0.75 ± 0.12	1.69 ± 0.37	1.52 ± 0.67

Values represent the means ± standard deviations (*n* = 3).

*p < 0.05, indicating a statistically significant difference between Transport_TC_, and Transport_MC_, or between Transport_ATP_, and Transport_AMP_., The final concentrations of positive substrates were 20 μM with incubation time of 10 min.

**TABLE 3:** Kinetic parameters for transports of Danshen phenolic acids by human and rat hepatic transporters.

Danshen phenolic acid	*K*_m_ (μM)	*V*_max_ (pmol/min/mg protein)	CL_int_ (μL/min/mg protein)
Human hepatic sinusoidal uptake OATP1B1			
Rosmarinic acid	1,371 ± 194	98.1 ± 5.9	0.07
Salvianolic acid D	403 ± 85	89.3 ± 7.4	0.22
Lithospermic acid	428 ± 67	27.0 ± 2.1	0.06
Salvianolic acid B	58.9 ± 6.2	28.1 ± 1.2	0.46
Human hepatic sinusoidal uptake OATP1B3			
Rosmarinic acid	750 ± 101	64.7 ± 3.1	0.09
Salvianolic acid D	570 ± 79	98.5 ± 4.8	0.17
Lithospermic acid	182 ± 26	9.59 ± 0.51	0.05
Salvianolic acid B	13.0 ± 1.6	11.0 ± 0.4	0.85
Human hepatic sinusoidal uptake OAT2			
Protocatechuic acid	185 ± 24	3,822 ± 177	20.7
Tanshinol	1,228 ± 160	7,475 ± 308	6.09
Rosmarinic acid	48.6 ± 4.2	927 ± 22	19.1
Salvianolic acid D	111 ± 13	2,687 ± 114	24.3
Rat hepatic sinusoidal uptake Oatp1b2			
Rosmarinic acid	2049 ± 418	159 ± 14	0.08
Salvianolic acid D	464 ± 62	572 ± 25	1.23
Salvianolic acid A	50.5 ± 12.8	19.2 ± 1.5	0.38
Lithospermic acid	91.3 ± 10.6	37.7 ± 1.4	0.41
Salvianolic acid B	20.5 ± 1.9	17.7 ± 0.5	0.87
Rat hepatic sinusoidal uptake Oat2			
Protocatechuic acid	109 ± 6	1,180 ± 19	10.9
Tanshinol	978 ± 123	1,437 ± 74	1.47
Rosmarinic acid	119 ± 15	620 ± 28	5.20
Salvianolic acid D	60.0 ± 7.2	164 ± 6	2.73

Values represent the means ± standard deviations (*n* = 3). Error estimates are based on the best fit of the average values obtained at each point to the Michaelis-Menten equation using nonlinear regression analysis.

Similar to the situation for human hepatic transporters, rosmarinic acid, salvianolic acid D, lithospermic acid, and salvianolic acid B were substrates of the rat hepatic sinusoidal uptake SLC transporter Oatp1b2 (the closest ortholog of both human hepatic OATP1B1 and OATP1B3) (Table 2); Figure 2 shows their saturable cellular uptake, with *K*_m_, *V*_max_, and CL_int_ values summarized in Table 3. Although neither protocatechuic acid nor tanshinol was a substrate of Oatp1b2, these two Danshen compounds, together with rosmarinic acid and salvianolic acid D, were substrates of Oat2 (another rat hepatic sinusoidal uptake SLC transporter), with their net transport ratios shown in Table 2 and associated *K*_m_, *V*_max_, and CL_int_ values in Figure 2 and Table 3. In addition, salvianolic acid B was a substrate of rat Mrp2, while rosmarinic acid and salvianolic acid D were substrates of rat Mrp4. Significant interspecies differences were observed for salvianolic acid A, as indicated by this compound being a substrate of Oatp1b2. No other rat hepatic transporter was found to mediate transport of the Danshen compounds.

### Transport of Danshen Phenolic Acids by Human and Rat Renal Transporters

**FIGURE 3:**
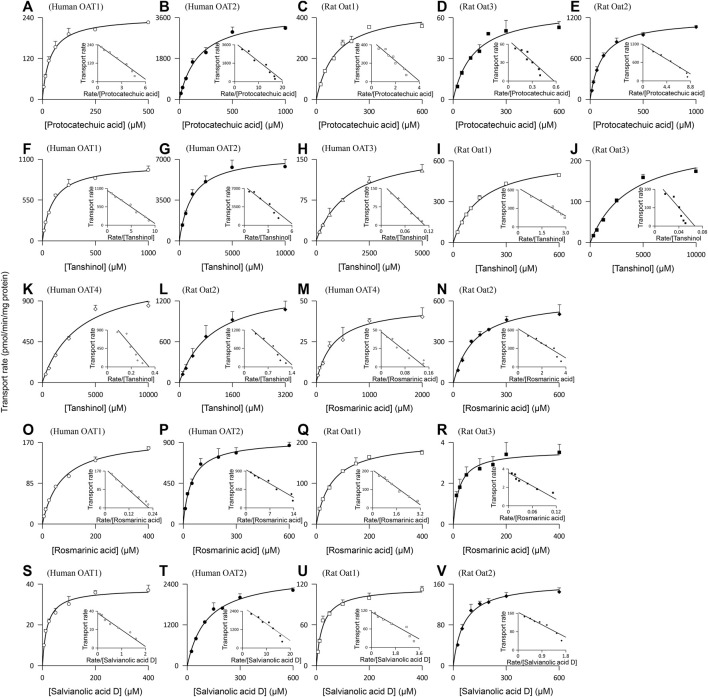
Representative kinetic plots of transport versus substrate concentration for cellular uptake of Danshen phenolic acids [**(A–E)**, protocatechuic acid; **(F–L)**, tanshinol; **(M–R)**, rosmarinic acid; and **(S–V)**, salvianolic acid D] mediated by the human and rat renal SLC transporters. The *K*_m_, *V*_max_, and CL_int_ values are shown in Table 5, and they represent the means ± standard deviations (*n* = 3). The final concentrations of the test Danshen compounds in the cellular uptake kinetic study are summarized in Table 1.

Protocatechuic acid (molecular mass, 154 Da), tanshinol (198 Da), rosmarinic acid (360 Da), and salvianolic acid D (418 Da) were found to be substrates of the human OAT1 and OAT2, two renal basolateral uptake SLC transporters. The compounds’ net transport ratios for OAT1 are shown in Table 4, while such data for OAT2 are shown in Table 2. Figure 3 shows their saturable cellular uptake for OAT1, with *K*_m_, *V*_max_, and CL_int_ values in Table 5; such data for OAT2 are shown in Figure 2 and Table 3. Recently, Kang et al. (2021) reported that rosmarinic acid was a substrate of human OAT1. In this investigation, we found that both OAT1 and OAT2 were responsible for cellular uptake of rosmarinic acid. Tanshinol and rosmarinic acid were also relatively weak substrates of human OAT4, a renal apical uptake SLC transporter, and OAT3 (tanshinol only), a renal basolateral uptake SLC transporter. The four Danshen phenolic acids were not substrates of other human renal transporters, except for salvianolic acid D being a substrate of renal MRP4. Regarding the other Danshen phenolic acids salvianolic acid A (molecular mass: 494 Da), lithospermic acid (538 Da), and salvianolic acid B (718 Da), no human renal basolateral uptake SLC transporter was found to mediate cellular transport of these compounds, albeit lithospermic acid and salvianolic acid B being substrates of the human renal apical efflux ABC transporter MRP2 (Table 4).

**TABLE 4:** Net transport ratios of Danshen phenolic acids at 100 μM final concentration by human and rat renal transporters.

Transporter	Transport_TC_/Transport_MC_ ratio for the SLC transporter or transport_ATP_/Transport_AMP_ ratio for the ABC transporter							
	Positive substrate	Protocatechuic acid	Tanshinol	Rosmarinic acid	Salvianolic acid D	Salvianolic acid A	Lithospermic acid	Salvianolic acid B
MW (Da)		154	198	360	418	494	538	718
Human renal basolateral uptake SLC transporters								
OAT1	50.2 ± 8.6 (PAH)	53.1 ± 3.9*	102 ± 26*	46.8 ± 10.9*	11.7 ± 2.4*	1.02 ± 0.14	2.09 ± 0.89	1.21 ± 0.14
OAT2	See OAT2 data in Table 2							
OAT3	10.2 ± 1.2 (E_1_S)	1.12 ± 0.25	4.54 ± 2.08*	1.56 ± 0.36	1.12 ± 0.56	2.12 ± 0.13	0.96 ± 0.15	1.03 ± 0.24
OCT2	10.0 ± 2.9 (TEA)	0.75 ± 0.35	1.11 ± 0.26	0.85 ± 0.29	0.85 ± 0.26	0.93 ± 0.32	0.89 ± 0.31	0.75 ± 0.23
Human renal apical uptake SLC transporters								
OAT4	7.69 ± 1.74 (E_1_S)	1.56 ± 0.46	7.81 ± 1.14*	7.27 ± 0.77*	2.87 ± 0.09	1.56 ± 0.23	2.91 ± 0.17	0.95 ± 0.23
PEPE1	126 ± 31 (GS)	0.93 ± 0.34	1.21 ± 0.51	1.42 ± 0.53	1.06 ± 0.20	1.45 ± 0.27	1.86 ± 0.36	2.05 ± 0.22
PEPT2	360 ± 49 (GS)	1.43 ± 0.32	1.12 ± 0.22	1.12 ± 0.22	1.03 ± 0.11	1.14 ± 0.31	0.74 ± 0.06	1.06 ± 0.12
Human renal apical efflux ABC transporters								
MRP2	See MRP2 data in Table 2							
MRP4	See MRP4 data in Table 2							
BCRP	See BCRP data in Table 2							
Rat renal basolateral uptake SLC transporters								
Oat1	70.5 ± 10.6 (PAH)	104 ± 20*	114 ± 18*	150 ± 46*	11.5 ± 2.9*	1.13 ± 0.64	1.36 ± 0.35	1.56 ± 0.67
Oat3	21.4 ± 6.8 (E_1_S)	16.1 ± 2.8*	5.83 ± 1.47*	4.45 ± 1.12*	1.41 ± 0.36	1.09 ± 0.35	0.94 ± 0.23	1.12 ± 0.41
Oct1	See Oct1 data in Table 2							
Oct2	7.49 ± 1.62 (TEA)	0.92 ± 0.08	1.09 ± 0.36	0.75 ± 0.32	1.44 ± 0.24	0.94 ± 0.33	1.11 ± 0.32	1.08 ± 0.21
Octn2	See Octn2 data in Table 2							
Rat renal apical uptake SLC transporters								
Oat2	See Oat2 data in Table 2							
Pept1	90.1 ± 10.4 (GS)	1.37 ± 0.36	1.14 ± 0.32	1.14 ± 0.13	1.43 ± 0.36	1.32 ± 0.13	1.31 ± 0.32	1.21 ± 0.45
Pept2	101 ± 19 (GS)	1.21 ± 0.26	1.19 ± 0.15	0.84 ± 0.17	1.88 ± 0.45	0.84 ± 0.19	0.85 ± 0.14	0.94 ± 0.21
Rat renal ABC apical efflux ABC transporters								
Mrp2	See Mrp2 data in Table 2							
Mrp4	See Mrp4 data in Table 2							
Bcrp	See Bcrp data in Table 2							

Values represent the means ± standard deviations (*n* = 3).

*p < 0.05, indicating a statistically significant difference between Transport_TC_, and Transport_MC_, or between Transport_ATP_, and Transport_AMP_., The final concentrations of positive substrates were 20 μM with incubation time of 10 min.

**TABLE 5:** Kinetic parameters for transports of Danshen phenolic acids by human and rat renal transporters.

Danshen phenolic acid	*K*_m_ (μM)	*V*_max_ (pmol/min/mg protein)	CL_int_ (μL/min/mg protein)
Human renal basolateral uptake OAT1			
Protocatechuic acid	37.6 ± 2.5	242 ± 4	6.43
Tanshinol	98.9 ± 7.1	1,038 ± 22	10.5
Rosmarinic acid	70.8 ± 6.4	181 ± 6	2.55
Salvianolic acid D	18.1 ± 2.3	37.6 ± 1.2	2.08
Human renal basolateral uptake OAT2			
Protocatechuic acid	See OAT2 data in Table 3		
Tanshinol	See OAT2 data in Table 3		
Rosmarinic acid	See OAT2 data in Table 3		
Salvianolic acid D	See OAT2 data in Table 3		
Human renal basolateral uptake OAT3			
Tanshinol	1,565 ± 127	171 ± 6	0.11
Human renal apical uptake OAT4			
Tanshinol	3,442 ± 940	1,204 ± 140	0.35
Rosmarinic acid	325 ± 53	47.5 ± 2.6	0.15
Rat renal basolateral uptake Oat1			
Protocatechuic acid	110 ± 19	446 ± 27	4.07
Tanshinol	154 ± 13	636 ± 22	4.13
Rosmarinic acid	59.2 ± 4.7	206 ± 5	3.47
Salvianolic acid D	24.4 ± 3.3	115 ± 4	4.72
Rat renal basolateral uptake Oat3			
Protocatechuic acid	117 ± 27	66.8 ± 5.5	0.57
Tanshinol	3,760 ± 907	252 ± 27	0.07
Rosmarinic acid	24.0 ± 4.2	3.58 ± 0.14	0.15
Rat renal apical uptake Oat2			
Protocatechuic acid	See Oat2 data in Table 3		
Tanshinol	See Oat2 data in Table 3		
Rosmarinic acid	See Oat2 data in Table 3		
Salvianolic acid D	See Oat2 data in Table 3		

Values represent the means ± standard deviations (*n* = 3). Error estimates are based on the best fit of the average values obtained at each point to the Michaelis-Menten equation using nonlinear regression analysis.

Similar to the situation for human renal transporters, protocatechuic acid, tanshinol, and rosmarinic acid were substrates of the rat renal basolateral uptake SLC transporters Oat1 and Oat3 (the closest orthologs of human renal OAT1 and OAT3, respectively) (Table 4); Figure 3 shows their saturable cellular uptake with *K*_m_, *V*_max_, and CL_int_ values in Table 5. Salvianolic acid D was a substrate of only rat Oat1, and not of rat Oat3. Unlike human OAT2 (a renal basolateral uptake SLC transporter), rat Oat2 is a renal apical uptake SLC transporter (Shen et al., 2015). Protocatechuic acid, tanshinol, rosmarinic acid, and salvianolic acid D were substrates of rat Oat2, while salvianolic acid A, lithospermic acid, and salvianolic acid D were not substrates of rat Oat1, Oat3, or Oat2. All the test Danshen compounds were not substrates of other rat renal transporters.

### New Insight Into Hepatobiliary and Renal Excretion in Concert for Elimination of Danshen Phenolic Acids in Humans and Rats

**FIGURE 4:**
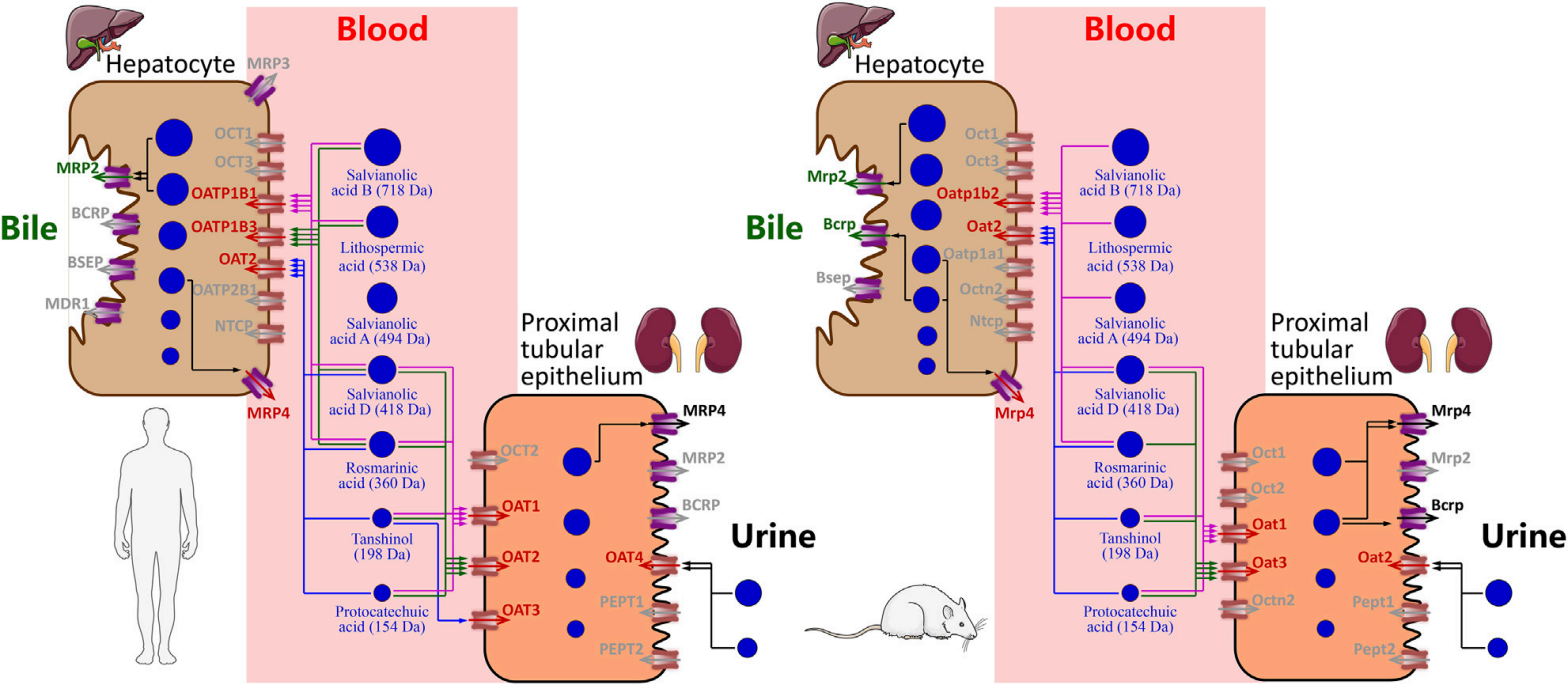
Schematic overview of mechanistic hepatobiliary and renal excretion of Danshen phenolic acids.

The Danshen phenolic acids protocatechuic acid (molecular mass, 154 Da), tanshinol (198 Da), rosmarinic acid (360 Da), salvianolic acid D (418 Da), salvianolic acid A (494 Da), lithospermic acid (538 Da), and salvianolic acid B (718 Da) are believed to be pharmacologically important for the injection due to their cardiovascular properties (Li et al., 2018; Ren et al., 2019). Because these herbal compounds are of poor or fairly good membrane permeability (Lu et al., 2008), their systemic elimination *via* the liver or kidneys necessitates transporter-mediated hepatic or renal uptake from blood, and this serves as the crucial elimination step. In this investigation, we found both hepatic and renal SLC transporters that could mediate uptake of the Danshen phenolic acids into the hepatocytes and/or renal proximal tubular epithelia, respectively, in a molecular-mass-related manner (Figure 4). The relatively high molecular mass compounds lithospermic acid and salvianolic acid B (both >500 Da) underwent systemic elimination, initiated by OATP1B1/OATP1B3-mediated hepatic uptake. Rosmarinic acid and salvianolic acid D (350–450 Da) underwent systemic elimination, initiated by OATP1B1/OATP1B3/OAT2-mediated hepatic uptake and by OAT1/OAT2-mediated renal uptake. The relatively low molecular mass compounds protocatechuic acid and tanshinol (both <200 Da) underwent systemic elimination, initiated by OAT1/OAT2-mediated renal uptake and OAT2-mediated hepatic uptake. However, unlike the preceding Danshen phenolic acids, little is known about how salvianolic acid A (494 Da) was taken up by the two organs. Given its molecular mass being around 500 Da, hepatic uptake of salvianolic acid A might be mediated by an unknown transporter(s). For protocatechuic acid, tanshinol, rosmarinic acid, and salvianolic acid D, the hepatic sinusoidal uptake SLC transporter OAT2 is important, by mediating the hepatic uptake, to facilitate hepatic metabolism (methylation, glucuronidation, and/or sulfation) of these Danshen phenolic acids of relatively low molecular mass (154–418 Da). Regarding the ABC transporters, hepatobiliary excretion of lithospermic acid and salvianolic acid B appeared to be accomplished by MRP2-mediated hepatic efflux into bile. Unlike these two high molecular mass compounds, no ABC transporters was found to mediate efflux of protocatechuic acid, tanshinol, rosmarinic acid, and salvianolic acid D into the bile and/or urine. Our earlier investigation by Tian et al. (2015) indicated that the methylated and sulfated metabolites of tanshinol could be extensively excreted, like the unchanged compound, into the urine. This suggested that involvement of metabolism in the excretion of the Danshen phenolic acids is worth considering. Given that protocatechuic acid and tanshinol have only fairly good membrane permeability and that permeability of rosmarinic acid and salvianolic acid D is poor, both accumulation of these compounds in the liver and/or kidneys and possibility of new ABC transporters that could mediate the compounds’ efflux merit further investigation. Regarding salvianolic acid D, the hepatic canalicular efflux ABC transporter MRP4 was found to be able to mediate the compound’s efflux into the blood; this could offset the OATP1B1/1B3-mediated uptake of the compound from the blood and in turn partially contribute the greatest systemic exposure to the compound after dosing DanHong (Li et al., 2015).

Drugs and their metabolites are usually eliminated *via* bile and/or urine. Understanding drug compounds’ tendency for excretion into the bile and/or urine is important, because the excretion can be a major factor influencing drugs’ therapeutic and toxicological properties. The belief that molecular mass influences the excretion tendency of a drug compound is implied in several early publications (Millburn, 1970; Smith, 1971; Yang et al., 2009). Drugs (particularly anions) highly excreted into bile tend to be of relatively high molecular mass (>500 Da); by contrast, drugs of relatively low molecular mass (<300 Da) tend to be excreted into the urine. Transporters play important roles in hepatobiliary and renal excretion of drugs (Morrissey et al., 2013; Liu and Sahi, 2016). However, little is known about how these transporters respond to drug difference in molecular mass. Our current investigation suggested that the human hepatic and renal uptake SLC transporters act on the Danshen compounds in a compound-molecular-mass-related manner, i.e., OATP1B transporters transporting those with molecular mass ≥360 Da, while OAT transporters transporting those with molecular masses ≤418 Da. A compound with a molecular mass of 360–418 Da could be taken up by both OATP1B and OAT transporters. More compounds of various types merit assessment for more precise setting of the molecular mass cutoffs for substrate preference of these SLC transporters.

For pharmacokinetic research on Chinese herbal medicines (Li, 2017; Zhang et al., 2018; Pintusophon et al., 2019; Lan et al., 2021; Li et al., 2021), rat is an experimental animal that is commonly used in supportive studies of herbal compounds to help understanding their important disposition steps, which are difficult to be measured in humans. To this end, it is important to gain insight into interspecies similarity and differences, before designing supportive animal studies. The hepatobiliary excretion of drugs varies widely among species; empirically, rat is a good biliary excreter for drugs (particularly for anions with molecular mass >320 Da), whereas human is a relatively poor excreter (Millburn et al., 1967; Lin and Lu, 1997). Similar to the human transporters, the rat hepatic and renal uptake SLC transporters act on the Danshen phenolic acids in a compound-molecular-mass-related manner. For these Danshen compounds, the molecular mass cutoffs for substrate preference of rat hepatic and renal SLC transporters were similar to those of the human transporters. In addition, the *K*_m_ values of the rat SLC transporters for the Danshen phenolic acids were generally comparable with those of their respective human orthologs. The observed interspecies difference was rat Oatp1b2 being able to mediate hepatic uptake of salvianolic acid A, whereas no human transporter was found that could do so. Collectively, PK similarities between human and rat, with respect to hepatic and renal disposition of the Danshen phenolic acids, makes the rat a suitable animal model for supportive studies as well as for associated pharmacodynamic studies, safety assessments, and drug-drug interaction (DDI) studies.

Transporters (particularly hepatic and renal uptake transporters) have been found to mediate potential pharmacokinetic DDIs with herbal medicines, comprising the herb-drug interaction (Meng and Liu, 2014; Jiang et al., 2015; Olaleye et al., 2019; Pintusophon et al., 2019) and the drug-herb interaction (Jia et al., 2015; Dong et al., 2018). The *in vitro* transporter kinetic data (Tables 3 and 5) indicated that most Danshen phenolic acids had low affinity for the human hepatic and renal uptake transporters with *K*_m_ values 38–3,442 μM, suggesting that these herbal compounds from DanHong were unlikely to induce herb-drug interactions by the transporters (DDI index, ≤ 0.02). Here, the DDI indices were estimated based on their (*C*_max_ × *f*_u-plasma_)/*K*_m_ ratios, where the *C*_max_ and *f*_u-plasma_ data were obtained from our earlier human pharmacokinetic study of DanHong (Li et al., 2015). Another investigation indicated that salvianolic acid B had a half maximum inhibitory concentration (IC_50_) of 38 µM for OATP1B1 (Li et al., 2019); the associated DDI index of 0.001 is still too low to induce DDI with normally dosed DanHong. Regarding the drug-herb interactions associated with the Danshen phenolic acids, our earlier investigation by Jia et al. (2015) indicated that tanshinol could be a victim of such interaction when OAT1/2/3 were inhibited by probenecid. Similarly, pharmacokinetic drug-herb interactions by inhibiting OAT1/2 need to be considered also for protocatechuic acid. Given that there are many dual OATP1B1/1B3 inhibitors in clinical use (Dong et al., 2018), pharmacokinetic drug-herb interactions *via* OATP1B1/1B3 inhibition could occur for lithospermic acid and salvianolic acid B, both of which underwent hepatic uptake but not renal uptake.

## Conclusion

In this investigation, we identified human transporters responsible for hepatobiliary and renal excretion of Danshen phenolic acids. Lithospermic acid and salvianolic acid B (both >500 Da) underwent systemic elimination, initiated by OATP1B1/OATP1B3-mediated hepatic uptake. Rosmarinic acid and salvianolic acid D (350–450 Da) underwent systemic elimination, initiated by OATP1B1/OATP1B3/OAT2-mediated hepatic uptake and by OAT1/2-mediated renal uptake. Protocatechuic acid and tanshinol (both <200 Da) underwent systemic elimination, initiated by OAT1/OAT2-mediated renal uptake and OAT2-mediated hepatic uptake. A similar scenario was observed with the rat orthologs, including the rat SLC transporters acting on the Danshen compounds, also in a compound-molecular-mass-related manner. The findings of this investigation improve our understanding of disposition of the Danshen phenolic acids and could facilitate pharmacokinetic research on other Danshen-containing injections. Future investigations are planned to elucidate whether any new transporter(s) could mediate hepatic uptake of salvianolic acid A and how efflux of many Danshen phenolic acids from hepatocytes and renal proximal tubular epithelia is achieved after the SLC transporter-mediated hepatic and renal uptake, respectively.

## Data Availability

The original contributions presented in the study are included in the article/Supplementary Materials, further inquiries can be directed to the corresponding authors.

## Abbreviations

ABC, ATP-binding cassette; BCRP/Bcrp, breast cancer resistance protein; BSEP/Bsep, bile salt export pump; CL_int_, intrinsic clearance; DDI, drug-drug interaction; E_1_S, estrone-3-sulfate; E_2_17βG, estradiol-17β-D-glucuronide; *f*_u-plasma_, unbound fraction in plasma; *K*_m_, Michaelis constant; GL, glycyrrhizin; GS, glycylsarcosine; MDR, multidrug resistance protein; MRP/Mrp, multidrug resistance-associated protein; MTX, methotrexate; NTCP/Ntcp, Na^+^/taurocholate cotransporting polypeptide; OAT/Oat, organic anion transporter; OATP/Oatp, organic anion-transporting polypeptide; OCT/Oct, organic cation transporter; Octn, carnitine/organic cation transporter; PAH, para-aminohippuric acid; PEPT/Pept, peptide transporter; PGF_2α_, prostaglandin F_2α_; SLC, solute carrier; TCA, taurocholic acid; TEA, tetraethylammonium; *V*_max_, maximum transport velocity.
