# Supplementary material for: Molecular Basis Underlying Hepatobiliary and Renal Excretion of Phenolic Acids of Salvia miltiorrhiza Roots (Danshen)
Source: Front Pharmacol. 2022 May 10;13:911982. doi: 10.3389/fphar.2022.911982 (PMC9127186; doi:10.3389/fphar.2022.911982)
Supplement: Supplementary file 1 [file DataSheet1.pdf]

Molecular Basis Underlying Hepatobiliary and Renal Excretion of Phenolic Acids of *Salvia miltiorrhiza* Roots (Danshen)

Jun-Lan Lu, Xue-Shan Zeng, Xin Zhou, Jun-Ling Yang, Ling-Ling Ren, Xin-Yu Long, Feng-Qing Wang, Olajide E. Olaleye, Nan-Nan Tian, Ya-Xuan Zhu, Jia-Jia Dong, Wei-Wei Jia, Chuan Li

Frontiers in Pharmacology (DOI: 10.3389/fphar.2022.911982)

Supplementary TABLE S1 | Danshen phenolic acids and protocatechuic aldhyde present in Danshen-containing injections approved by Chinese NMPA for medicinal use.

Danshen-containing injection	Protocatechuic aldhyde	Protocatechuic acid	Tanshinol	Rosmarinic acid	Salvianolic acid D	Salvianolic acid A	lithospermic acid	Salvianolic acid B
	μmol/day							
Danshen injection	52.2	0.6	127.5	13.9	3.8	5.5	2.3	8.8
Freeze-dried Danshen	49.4	0.8	237.9	20.3	14.5	22.1	3.9	3.0
Freeze-dried Salvianolic acid	0.4	0.01	2.0	31.2	0.5	0.8	3.1	234.8
DanHong injection	161.4	4.3	679.0	72.9	53.6	84.8	10.2	111.9
GuanXinNing injection	144.2	3.9	417.6	55.0	34.3	19.5	17.2	65.4
XiangDan injection	78.3	1.5	160.5	22.9	7.4	3.4	2.9	8.3
ShenKang injection	12.3	0.5	61.1	4.8	6.8	2.2	0.8	3.0
XueBiJing injection	43.7	2.2	35.8	19.1	0.9	1.2	0.9	6.8
DanshenChuanQiongQin injection	6.4	0.04	18.3	1.4	1.6	0.9	0.2	0.3

An liquid chromatography/mass spectrometry-based composition analytical assay (Li et al., 2015) was used in analysis of samples of the Danshen-containing injections.

REFERENCE

Li, M., Wang, F., Huang, Y., Du, F., Zhong, C., Olaleye, O. E. et al. (2015). Systemic Exposure to and Disposition of Catechols Derived from *Salvia Miltiorrhiza* Roots (Danshen) after Intravenous Dosing Danhong Injection in Human Subjects, Rats, and Dogs. *Drug Metab Dispos* 43, 679-690. doi: 10.1124/dmd.114.061473
